# Low-Dose Ibutilide Combined with Catheter Ablation of Persistent Atrial Fibrillation: Procedural Impact and Clinical Outcome

**DOI:** 10.1155/2019/3210803

**Published:** 2019-01-02

**Authors:** Xue-Rong Sun, Ying Tian, Ashok Shah, Xian-Dong Yin, Liang Shi, Yan-Jiang Wang, Xiao-Qing Liu, Meleze Hocini, Michel Haissaguerre, Xin-Chun Yang, Xing-Peng Liu

**Affiliations:** ^1^Heart Center, Beijing Chao-Yang Hospital, Capital Medical University, Beijing 100020, China; ^2^Service de Rhythmologie, Hopital Cardiologique du Haut-Lévêque, Bordeaux 33000, France

## Abstract

**Background:**

In patients with persistent atrial fibrillation (AF), the procedural and clinical outcomes of ablation combined with infusion of antiarrhythmic drug are unknown.

**Objectives:**

To determine the impact of low-dose ibutilide after circumferential pulmonary vein isolation (CPVI) and/or left atrial (LA) substrate modification on acute procedural and clinical outcome of persistent AF.

**Methods:**

In a prospective cohort of 135 consecutive patients with persistent AF, intravenous 0.25 mg ibutilide was administered 3 days before the procedure and intraprocedurally, if required, after CPVI and/or additional LA substrate modification of sites with continuous, rapid or fractionated, and low-voltage (0.05–0.3 mv) atrial activity.

**Results:**

Persistent AF was terminated by CPVI alone (*n*=15) or CPVI + ibutilide (*n*=32) in 47 (34.8%) patients (CPVI responders). Additional LA substrate modification without (*n*=33) or with subsequent administration of 0.25 mg ibutilide (*n*=19) terminated AF in another 52 (38.5%) patients (substrate modification responders). Sinus rhythm was restored by electrical cardioversion in the remaining 36 (26.7%) patients (nonresponders). The mean LA substrate ablation time was 14 ± 6 minutes. At follow-up of 24 ± 10 months, the rates of freedom from atrial tachyarrhythmias among the responders in CPVI and substrate modification groups were mutually comparable (66.0% and 69.2%) and higher than among the nonresponders (36.1%; *P* < 0.01). Among the responders, there was no difference in clinical outcome between patients whose persistent AF was terminated without or with low-dose ibutilide.

**Conclusion:**

Administration of low-dose ibutilide during ablation of persistent AF may allow select patients wherein substrate ablation is not or minimally required to optimize procedural and clinical outcomes.

## 1. Introduction

Persistent atrial fibrillation (AF) represents a major challenge in catheter ablation of arrhythmias [1]. Although various strategies have been proposed [2–6], the long-term efficacy of ablation remains disappointingly low, particularly in long-standing persistent AF [7–9]. Thus, there is an important unmet clinical need for an individualized and a truly substrate-based approach for ablation of persistent AF.

Recent studies using novel technologies highlighted the diversity of the left atrial (LA) substrate and the importance of specific atrial areas, harboring rotor and focal impulses, in maintaining persistent AF [10, 11]. It is worth noting that these technologies, at their current stage of development, are not widely diffused. Using conventional mapping techniques, on the other hand, it is difficult to identify the key atrial substrate of persistent AF because of the chaotic pattern of atrial activity in most patients. However, this raises an important question: can these key atrial substrates be revealed by conventional mapping potentiated by drugs which have little effect on AF drivers but significantly reduce bystander atrial activities? In a canine model of pacing-induced sustained AF, Chou et al. found that ibutilide had significant effects on reentrant wave fronts in the pulmonary vein (PV) and PV-LA junction but did not suppress the fast, repetitive, and rapid activities responsible for the maintenance of AF [12]. In addition, a study that included 11 patients with persistent AF suggested that intraprocedural administration of low-dose ibutilide after circumferential PV isolation (CPVI) was effective in organizing AF activity [13]. Yet, the long-term outcomes in persistent AF patients terminated by ablation with or without infusion of low-dose ibutilide remain unknown.

We conducted a prospective study aiming to test three hypotheses: (1) low-dose ibutilide could facilitate acute termination of persistent AF; (2) ibutilide could help to discriminate AF drivers from passive bystander activities and thereby limit ablation of the atrial substrate after CPVI; and (3) acute AF termination by this combined approach could be associated with favorable long-term outcome.

## 2. Methods

### 2.1. Study Cohort

The study cohort consisted of 139 consecutive patients who were referred for catheter ablation of symptomatic persistent AF refractory to antiarrhythmic drugs. The inclusion criteria were age between 18 and 80 years, nonvalvular AF, no previous catheter ablation of AF, and availability of informed consent. Patients with any of the following characteristics were excluded from this study: AF with a reversible cause (such as hyperthyroidism); LA diameter ≥55 mm on echocardiography; New York Heart Association class IV heart failure, left ventricular ejection fraction less than 35%, and severe hypertrophic cardiomyopathy (ventricular septal thickness >20 mm); and a baseline-corrected QT interval ≥480 ms. Persistent and long-lasting persistent AF were defined as continuous AF that persisted for >1 week and >1 year, respectively. The study protocol was approved by the institutional review board.

### 2.2. Preablation Preparation

All antiarrhythmic drugs were discontinued for at least 5 half-lives, and amiodarone was stopped for at least 2 months prior to ablation. Warfarin was discontinued 3–5 days before ablation and was replaced with subcutaneous injections of low-molecular-weight heparin. Transesophageal echocardiography and cardiac-computed tomography were performed 1 day before the procedure to exclude LA thrombi and reconstruct 3-dimensional anatomy of the PVs and LA. Three days before the procedure, 0.25 mg ibutilide was injected intravenously over 3 minutes, and rhythm was monitored for 24 hours in all patients. If persistent AF was terminated by this low dose of ibutilide, the patient was excluded from the study.

### 2.3. Electrophysiological Study

The procedure was performed under conscious sedation using fentanyl and midazolam, as required. A steerable decapolar catheter (XT™, Bard Electrophysiology, Lowell, MA, USA) was positioned in the coronary sinus via the left femoral vein. After transseptal catheterization, two long sheaths (SL1, St. Jude Medical, MN, USA) were advanced into the LA and flushed with continuous injection of saline (20 mL/h) to avoid thrombus formation or air embolism. During the whole procedure, the activated clotting time was maintained between 250 and 300 seconds by intravenous administration of heparin. The surface electrocardiogram (ECG) and bipolar endocardial electrograms were continuously monitored and recorded with a computer-based digital amplifier and recording system (Bard Electrophysiology, Lowell, MA, USA).

After PV angiography, the AF cycle length (AFCL) was recorded at baseline within each PV and left atrial appendage (LAA) using a decapolar circular catheter (Lasso™, Biosense Webster, Diamond Bar, CA, USA). Three-dimensional electroanatomical LA reconstruction was performed by using the CARTO 3 system (Biosense Webster, Diamond Bar, CA, USA), and ablation was performed with a 3.5 mm-tip irrigated catheter (TheromoCool NaviStar, Biosense Webster, Diamond Bar, CA, USA).

### 2.4. Ablation Protocol

**Figure 1:**
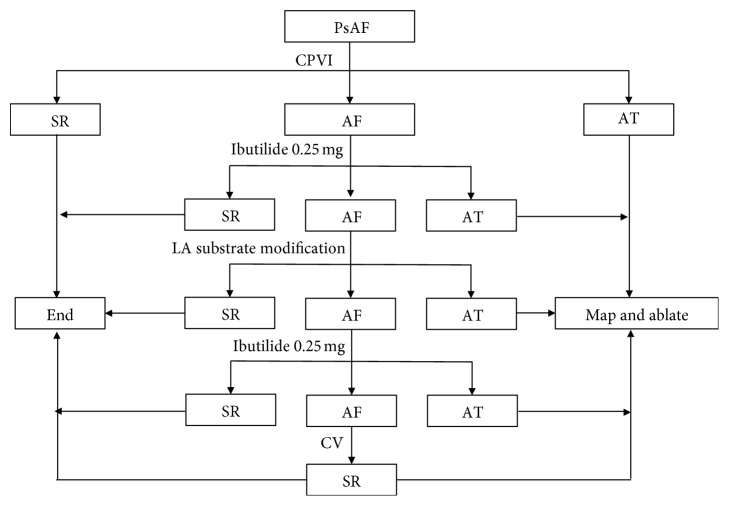
The ablation protocol used in this study. PsAF = persistent atrial fibrillation; SR = sinus rhythm; AT = atrial tachycardia; LA = left atrial; CV = cardioversion.

The ablation protocol is shown in Figure 1. Briefly, the first step was CPVI in all patients. If persistent AF converted to sinus rhythm (SR) during CPVI, no further ablation was performed. If AF continued, 0.25 mg ibutilide was administered over 3 minutes, and AFCL within the LAA was recorded every 5 minutes, thereafter. If AF converted to SR within a 30-minute period, no further ablation was performed. In patients with SR, a voltage map of the LA was created and the percentage of areas with low-amplitude (<0.5 mV) atrial signals was calculated. When AF continued, the LA substrate was ablated (see descriptions below) until it terminated or until the total ablation time reached 30 minutes, whichever was earlier. In the latter situation, a second dose of 0.25 mg ibutilide was injected over 3 minutes, and the patient was observed for another 30 minutes. Unless AF was terminated within 30 minutes, SR was restored by electrical cardioversion. If AF converted to AT anytime during the procedure, it was mapped and ablated until SR was restored. Finally, the completeness of the 2 circular lines each surrounding the ipsilateral PVs was checked by bolus injection of 20 mg adenosine triphosphate in all patients. If a patient received any atrial linear lesions for macro-reentrant ATs, bidirectional conduction block across the lesions was confirmed by using the differential pacing maneuver.

Radiofrequency energy was delivered from the Stockert generator at a temperature setting of 43°C, with power limited to 30 to 35 W at an irrigation speed of 17 mL/min in the LA and to 20 to 25 W at an irrigation speed of 30 mL/min within the coronary sinus.

### 2.5. Target Sites for LA Substrate Modification

**Figure 2:**
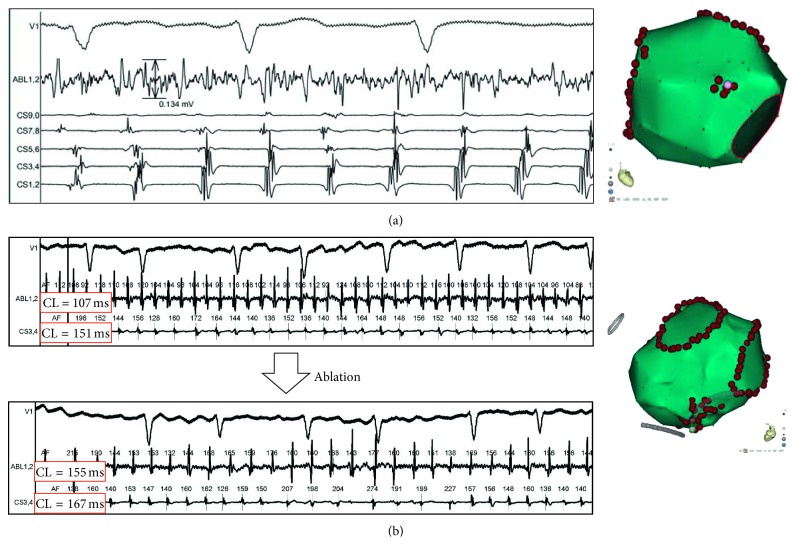
Examples of ablation targets. (a) Surface ECG lead V1, intracardiac recordings from an ablation catheter (abl), and a mapping catheter within the coronary sinus (CS). Note that, after circumferential pulmonary vein isolation (CPVI) and ibutilide, the atrial activity with CS had become very organized; however, an area on the anterior wall of the left atrium (LA) still presented with continuous, low-voltage (0.134 mV), and fractionated atrial electrograms. (b) Surface ECG lead V1 and intracardiac recording from an ablation catheter and CS 3-4 electrodes. After CPVI and ibutilide, the local atrial fibrillation cycle length (AFCL) was 107 ms on the lower part of the posterior wall of the LA, whereas the AFCL recorded by the adjacent CS catheter was more rapid (151 ms) (top). After ablation, the local AFCLs at the ablation site and CS were prolonged to 155 and 167 ms, respectively (bottom).

The sites with the following characteristics were tagged and ablated: (a) continuous (>5 s), low-amplitude (0.05–0.3 mV), and fractionated atrial electrograms [3] and (b) local atrial deflections more rapid than those from the adjacent sites (Figure 2).

### 2.6. Repeat Ablation Procedure

If an atrial tachyarrhythmia recurred, ablation was repeated at least 3 months after the index procedure. During the repeat procedure, the PVs were checked for recovery of conduction and reisolated if they were reconnected. Isoproterenol and/or adenosine triphosphate were used to trigger non-PV foci in patients with recurrent paroxysmal AF. If the recurrent AF was unrelated to PV, the LA substrate was ablated during the repeat procedure. All recurrent ATs were also mapped and ablated.

### 2.7. Postprocedural Care and Follow-Up

Antiarrhythmic medications were resumed postablation, and patients were monitored inhospital for 2 to 3 days prior to discharge. Arrhythmias recurring within a 3-month blanking period after ablation were cardioverted, if required. Follow-up at the outpatient clinic was scheduled for 2 weeks and 1, 3, 6, 9, and 12 months after the procedure and 6 monthly thereafter. Twelve-lead ECG was obtained at each visit, and serial 72-hour Holter was undertaken at 6, 12, 18, and 24 months. Additional 24-hour Holter or ECG was undertaken in patients reporting with symptoms. Recurrence was defined as documented AF/AT lasting >30 s after the 3-month blanking period.

Postprocedural anticoagulation was continued for 6 months in the absence of arrhythmia recurrence or for a longer period based on the CHA_2_DS_2_-VASc score. Repeat ablation was encouraged in patients with recurrent AF/AT beyond the blanking period.

### 2.8. Statistics

All data are reported as a mean ± SD for continuous variables and number of subjects (%) for categorical variables unless otherwise indicated. For baseline demographics and procedure parameters, continuous variables were compared using ANOVA with the modality by which SR was achieved during the procedure as the factor and categorical variables were compared using Fisher's exact test. Changes in AFCL were compared using paired *T*-test. Freedom from atrial tachyarrhythmias was determined and compared using the Kaplan–Meier analysis and the log-rank test. To identify independent predictors of termination of AF during ablation and of clinical success, multivariate logistic regression was conducted, with only covariates. All hypotheses were 2-sided tests with a type I error level set to 0.05.

## 3. Results

### 3.1. Patient Characteristics

Among consecutive 139 eligible patients, administration of ibutilide 3 days before the ablation procedure terminated persistent AF in 4 (2.8%), who were, therefore, excluded from the study. The baseline characteristics of the remaining 135 patients are presented in Table 1.

### 3.2. Acute Outcome of CPVI

The baseline AFCL within the LAA, left superior PV, left inferior PV, right superior PV, and right inferior PV were 148 ± 21 ms, 147 ± 22 ms, 145 ± 19 ms, 151 ± 24 ms, and 147 ± 21 ms, respectively. All PVs were isolated during AF. During CPVI, persistent AF converted directly to SR in 7 patients (5.2%) and organized to typical atrial flutter in 8 patients (5.9%). AF persisted in the remaining 120 (88.9%) patients. Pre- and post-CPVI and AFCLs within the LAA were similar (148 ± 21 ms vs. 149 ± 20 ms, *P*=0.689).

### 3.3. Effect of Intraprocedural Ibutilide after CPVI

After CPVI, persistent AF was converted into SR in 27/120 patients and organized to AT in 5/120 patients within 30 minutes of ibutilide infusion. In the remaining 88/120 (73.3%) patients, LAA-AFCL was significantly prolonged. The AFCL prolongation started at the end of the infusion (0 min, 161 ± 23 ms, *P* < 0.05) and peaked at 5 or 10 minutes later (173 ± 23 and 173 ± 24 ms, respectively; paired *T*-test *P* < 0.05 for both). It shortened insignificantly thereafter within 30 minutes.

### 3.4. LA Voltage Map in SR

An LA voltage map in SR was obtained for 39/47 patients in whom persistent AF was terminated by CPVI alone or combined with ibutilide. Low-voltage (<0.5 mV) areas were found in 6 patients, covering mean 15 ± 6% of the LA surface. In others, the LA voltage map did not show any area of low voltage.

### 3.5. Acute Outcome of LA Substrate Ablation without and with Ibutilide

**Figure 3:**
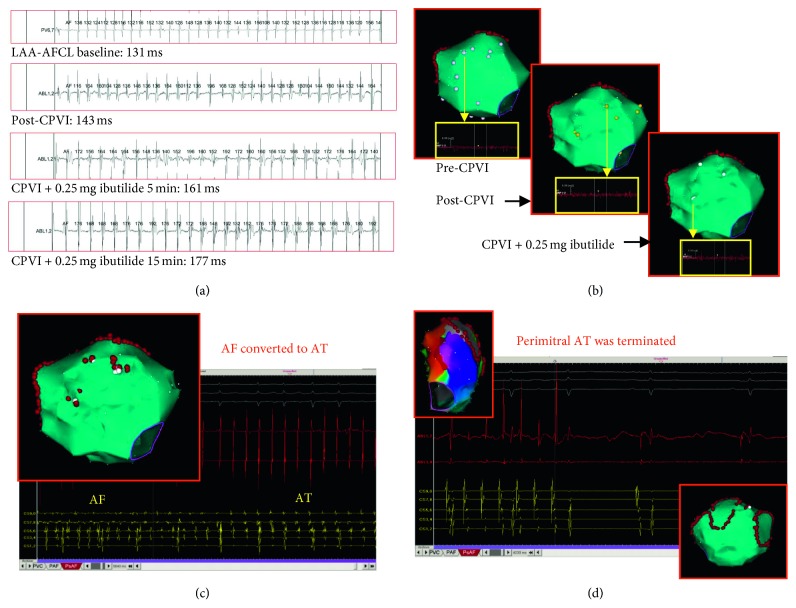
An example of persistent atrial fibrillation (AF) ablation with this low-dose ibutilide-guided approach. (a) The AF cycle length (AFCL) within the left atrial appendage (LAA) at baseline, after circumferential pulmonary vein isolation (CPVI), and 5 and 15 minutes after administration of 0.25 mg ibutilide. (b) Three electroanatomic maps showing the sites where low-amplitude complex fractionated atrial electrograms (CFAEs) were recorded on the anterior wall of the left atrium (LA). The pink, yellow, and white points represent CFAEs recorded before CPVI, after CPVI, and after administration of 0.25 mg ibutilide, respectively. The CFAE areas were minimized after CPVI plus ibutilide. (c) Ablation targeting these areas converted AF to atrial tachycardia (AT). (d) The 3D electroanatomic mapping of LA suggested that the mechanism of this AT was perimitral reentrant tachycardia (left upper), and ablation (alone) at the mitral isthmus terminated this AT.

**Figure 4:**
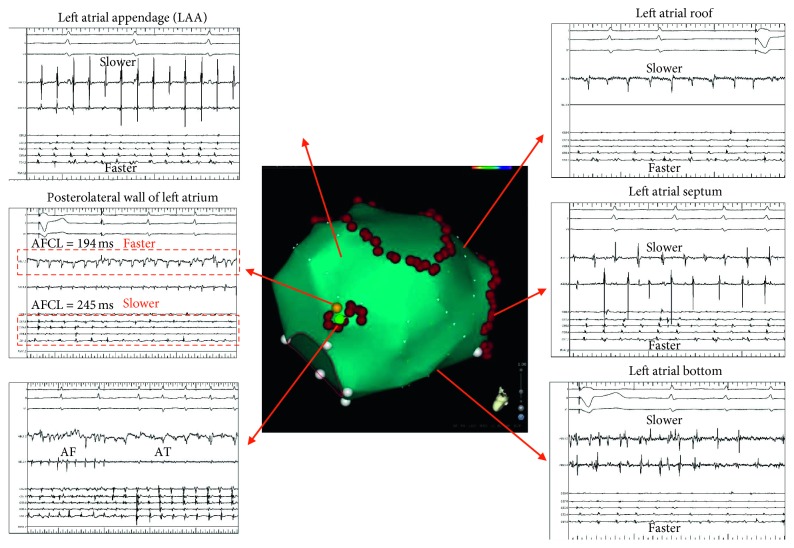
Ablation of the site with the most rapid atrial fibrillation cycle length (AFCL) uncovered by low-dose ibutilide administration terminated persistent AF. The middle figure is the 3D electroanatomic map of the left atrium (LA) in this patient. The red points represent the lesions surrounding the PV antrum. After administration of 0.25 mg ibutilide, the LA activity became organized, and the AFCL could be measured at most of the sites in the LA. From top to bottom on the right panel are the intracardiac recordings (abl) from the LA roof, septum, and bottom. The activity at these sites and in the LA appendage (left upper) was longer than that in the coronary sinus. However, the AFCL at the posterolateral aspect of the LA (left middle, yellow point) was significantly shorter than that in the CS (194 vs 245 ms), suggesting that this area was harboring an AF driver. During regional ablation of this small region, AF converted to atrial tachycardia (AT, green point).

Ablation terminated persistent AF in 33/88 (37.5%) patients, restoring SR directly in 12/88 (13.6%) patients and via intermediate AT in 21/88 (23.9%) patients. The AF termination sites were posteroinferior LA (*n*=10), LA septum (*n*=6), LAA (*n*=4), anterior LA (*n*=4), posterior LA (*n*=3), lateral LA (*n*=3), and LA roof (*n*=3). Figures 3 and 4 show 2 different examples of LA substrate ablation leading to termination of AF. In 55 (62.5%) patients, whose AF persisted after 30 minutes of LA substrate ablation, a second bolus of 0.25 mg ibutilide restored SR in 11 (12.5%) patients and converted AF to AT in 8 (9.1%) patients. AF was electrically cardioverted in the remaining 36 (40.9%) patients.

In total, intraprocedural AF termination by ablation ± ibutilide was achieved in 99/135 (73.3%) patients. The ablation times for CPVI and LA substrate modification were 28 ± 10 min and 14 ± 6 min, respectively. Other procedural parameters are summarized in Table 2.

### 3.6. Mapping and Ablation of Intermediate ATs

Totally, 49 ATs were encountered in 46 (34.1%) patients. The most common AT was cavotricuspid isthmus-dependent flutter (*n*=34, 57.6%), followed by LA roof-dependent flutter (*n*=10, 16.9%), perimitral flutter (*n*=10, 16.9%), and focal AT (*n*=5, 5.7%). All but 4 ATs were terminated by ablation. Cardioversion was performed in 4 patients with perimitral flutter, and further ablation of mitral isthmus was performed during pacing from the distal coronary sinus. The bidirectional block across the linear lesions was achieved in all patients who underwent linear ablation.

### 3.7. Long-Term Clinical Outcome

At 12 months and 24 ± 10 months after the index procedure, freedom from atrial tachyarrhythmias off antiarrhythmic drugs was achieved in 98 (72.6%) and 80 (59.3%) patients, respectively. Recurrent arrhythmias included persistent AF, paroxysmal AF, persistent AT, and persistent AF alternating with AT in 18, 15, 7, and 15 patients, respectively.

The study population was divided into 3 groups as follows: CPVI responders (SR/AT following CPVI without or with ibutilide, *n*=47), LA substrate responders (SR/AT following LA substrate ablation without or with ibutilide, *n*=52), and nonresponders (electrical cardioversion of AF, *n*=36). The clinical characteristics of each of these groups are shown in Table 1. The duration of persistent AF and the LA volume were significantly greater in the nonresponders than in the responders, suggesting a more severe arrhythmogenic substrate in nonresponders.

**Figure 5:**
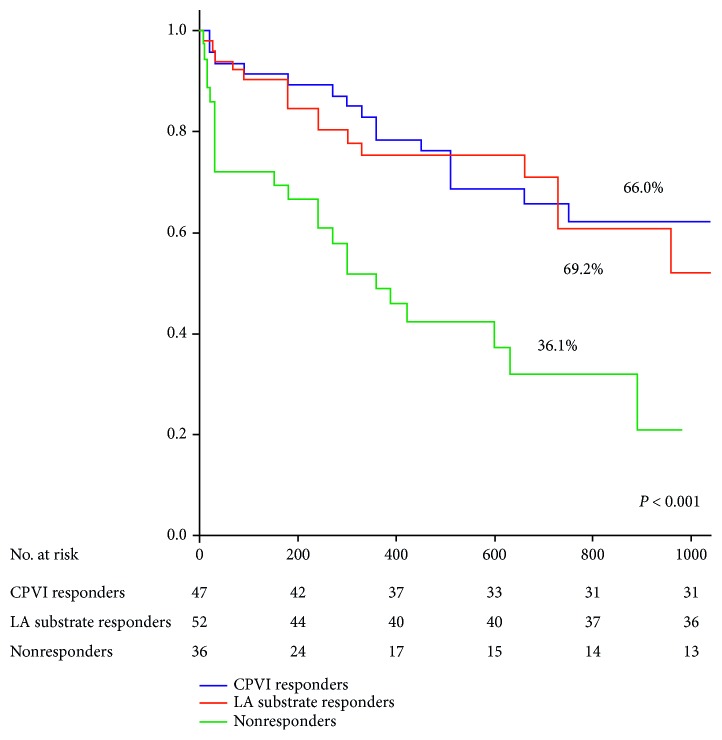
Kaplan–Meier plots depicting atrial tachyarrhythmia-free survival at 2 years after the first procedure. Persistent atrial fibrillation (AF) was terminated by circumferential pulmonary vein isolation (CPVI) alone (blue line), CPVI combined with left atrial modification guided by low-dose ibutilide (red line), and cardioversion (CV) (green line).

The 24-month success rates were comparable between the CPVI and LA substrate responders (66% versus 69.2%, *P*=0.830), but significantly higher than that of the nonresponders (36.1%, *P* < 0.001, Figure 5). Among the CPVI responders, the 2-year success rates did not differ between the patients who received and who did not receive intraprocedural ibutilide (62.5% versus 73.3%, *P*=0.528). Similarly, in the LA substrate responders' group, the 2-year success rates did not differ between the patients who received and who did not receive ibutilide (73.7% versus 66.7%, *P*=0.758).

### 3.8. Redo Procedures and Outcome

A total of 23/135 (17%) patients underwent a second ablation procedure, and 1 of them had 3 ablation procedures. After the last procedure, 14/23 (60.9%) patients were in stable SR at mean 21 ± 15 months of follow-up.

### 3.9. Periprocedural Complications

A severe left hemothorax occurred in 1 patient just after ablation. Thoracocentesis relieved the symptoms without any sequelae. Hematoma in the right groin was observed in 4 (3%) patients. Ibutilide induced premature ventricular complexes with right bundle branch block morphology during the procedure in 20 (14.8%) patients but no ventricular tachycardia up to 24 hours after procedure. No cardiac tamponade, stroke, or atrial-esophageal fistula were observed.

### 3.10. Predictors of Long-Term Outcome after the Index Ablation Procedure

Multivariate logistic regression analysis identified small left atrial volume (OR 0.976, 95% CI 0.956–0.996, *P*=0.020) and intraprocedural AF termination (OR 7.675, 95% CI 2.362–24.943, *P*=0.001) as the independent predictors of long-term outcome after the index ablation procedure.

## 4. Discussion

In this study, we systematically investigate the role of intraprocedural administration of low-dose ibutilide during catheter ablation for persistent AF. It confirms that (1) intraprocedural termination of persistent AF is associated with a favorable long-term outcome and demonstrates that low dose of ibutilide may extend important benefit and (2) the long-term outcome was similar between patients in whom AF was terminated by ablation alone or in combination with intraprocedural ibutilide.

### 4.1. Use of Low-Dose Ibutilide in Persistent AF Ablation

The clinical indication for administering ibutilide is recent-onset AF or atrial flutter with a routine dose of 1 to 2 mg [14]. In a dose-response study, Ellenbogen et al. found that AF termination could only be achieved in 12% of such patients if a very low dose (0.005 mg/kg) of ibutilide was used [15]. In the current study, the AF termination rate using 0.25 mg (≈0.004 mg/kg for a body weight of 60 kg) of ibutilide, 3 days before the procedures, was only 2.9% (*n*=4). Of note, the AF was not recent-onset in this study. Interestingly, when the same dose of ibutilide was used intraprocedurally again after 3 more days (beyond >5 half-lives (2–12, mean 6 hours)), the AF termination rate increased to 23.7% (*n*=32) after CPVI and additionally by 14.1% (*n*=19) after both CPVI and LA substrate modification. These results suggest that ablation can modify the atrial substrate facilitating termination of persistent AF with low-dose ibutilide.

### 4.2. Acute Intraprocedural AF Termination and Long-Term Outcome

In line with most studies of persistent AF ablation, this study showed a clear difference in long-term outcome between patients with and without intraprocedural AF termination [7]. Failure to terminate persistent AF during ablation could indicate that the critical substrate was not being targeted by ablation most likely because it was not identified [16, 17]. On the other hand, ablation targeting those sites presenting with low-dose ibutilide refractory atrial activities which can acutely terminate persistent AF in 59.1% (52/88) of patients suggests that these sites are potentially the areas harboring drivers of persistent AF. Also, for patients treated with either CPVI or CPVI plus LA substrate ablation, an important finding of this study is that the long-term outcome was similar between patients in whom AF was terminated by ablation or by ablation combined with ibutilide. This means that the atrial substrate “erased” by low-dose ibutilide was not critical to the maintenance of persistent AF. This role of ibutilide in discriminating AF drivers from passive bystander activities raises an intriguing hypothesis, where ibutilide may be considered to affect preferentially a functional and reversible part of the substrate, such as electrical remodeling. In this study, the mean ablation time taken for LA substrate modification was only 14 minutes, suggesting that the combined approach limited the atrial damage. It underscores the desirable influence of ibutilide in limiting the ablation of the atrial substrate after CPVI.

### 4.3. Identification of Patients in Whom CPVI May Be Enough

Recently, the STAR AF II trial reported that a CPVI alone approach is effective in 41% of patients with persistent AF [18]. The question, however, is how to identify these patients intraprocedurally. In the present study, CPVI without and with ibutilide terminated persistent AF in 1/3 of study population, and more importantly, the follow-up data, showed that the rate of freedom from atrial tachyarrhythmias among these patients was 83% at 12 months and 66% after 2-year-long follow-up, which supports the hypothesis that further LA substrate ablation is not be required in this subset of patients. Notably, in the CPVI responders' group, there was no difference in the 2-year success rate between patients whose PsAF was terminated without or with ibutilide, suggesting that low-dose ibutilide avoided LA substrate ablation in 32 more patients actually.

### 4.4. Localization of the Key LA Substrate by Using Low-Dose Ibutilide

Considering that there is substantial variability in the characteristics of atrial substrate and that the majority of complex fractionated atrial electrogram are actually functional [19], an individualized approach targeting the key atrial drivers, such as rotors and foci, has gained interest recently [10, 11]. Although the ability of conventional bipolar electrograms to diagnose such key driver locations remains subpar, some of the characteristics have been regarded as indicators of driver activity [20]. In the current study, we found that 0.25 mg ibutilide significantly prolonged the AFCL by organizing atrial electrograms in several areas, possibly the bystander sites. The sites presenting continuous, rapid or low-voltage, and fractionated activity were regarded as key locations of AF drivers. Identification of limited atrial sites with characteristic signals as ablation targets favourably reduced the extent and duration of substrate ablation to achieve AF termination.

### 4.5. Limitations

There are three main limitations of this study. First, the right atrium was not mapped after administration of ibutilide. Recent studies have shown that the right atrium harbors about 30% of drivers of persistent AF [12, 13]. Second, this is a single-center study and its findings need to be reproduced in a multicenter trial. However, its sample size is quite large, and the duration of follow-up is aptly long. Currently, such a multicenter study utilizing biatrial mapping is underway. Finally, we only monitored the AFCL in the LAA after CPVI. If we also checked the AFCL in other atrial sites simultaneously, such as right atrial appendage, the following atrial substrate mapping process may be facilitated.

## 5. Conclusion

In patients undergoing ablation of persistent AF, intraprocedural use of low-dose ibutilide may allow select patients in whom additional substrate ablation after CPVI can be avoided or reduced without compromising the long-term clinical success.

## 6. Clinical Perspectives

During catheter ablation of persistent atrial fibrillation (AF), it is difficult to identify the key atrial substrate of AF because of the chaotic pattern of atrial activity in most patients. In this prospective study enrolling 135 patients with persistent AF, we have systematically tested the hypotheses that intraprocedural low-dose (0.25 mg) ibutilide would distinguish between the patients who will do well just with circumferential pulmonary vein isolation (CPVI) versus those who would require additional atrial substrate ablation. In the latter group, low-dose ibutilide could unravel the key AF driver locations from passive bystander sites. Persistent AF was terminated in 99/135 (73.3%) patients using the combination of “ablation + ibutilide” approach. Notably, left atrial ablation was not required in 47 (34.8%) patients to terminate AF and in other 52 (38.5%) patients, where it was required, and its duration was reduced to mean 14 minutes. After 2-year-long follow-up, freedom from atrial tachyarrhythmias was similar between 52 patients who required it and 47 patients, who did not (69.2% and 66%). In patients wherein the combination approach did not result in AF termination (*n*=36), the long-term arrhythmia freedom was significantly worse (36.1%; *P* < 0.01). Thus, combining the two may allow selection of a large subset of patients in whom CPVI is good enough and atrial ablation is unnecessary. Also, in patients who remain to be the candidates for atrial ablation, the potentiating effect of ibutilide on ablation facilitates AF termination with minimal duration of atrial ablation. All acute effects of ibutilide-facilitated ablation translate similarly into long-term clinical outcome.

## Figures and Tables

**Table 1:** Patient characteristics.

	Total (*n*=135)	CPVI responders (*n*=47)	LA substrate responders (*n*=52)	Nonresponders (*n*=36)	*P* value
Age, years	63 ± 10	62 ± 12	63 ± 9	63 ± 10	0.913
Male, *n* (%)	85 (63.0)	26 (55.3)	31 (59.6)	28 (77.8)	0.090
BMI	26 ± 5	26 ± 5	26 ± 5	27 ± 3	0.873
Hypertension, *n* (%)	95 (70.4)	29 (61.7)	38 (73.1)	25 (69.4)	0.470
Underlying heart disease, *n* (%)	25 (18.5)	9 (19.1)	12 (23.1)	4 (11.1)	0.361
Diabetes mellitus, *n* (%)	31 (23.0)	6 (12.8)	18 (34.6)	7 (19.4)	0.030
History of stroke, *n* (%)	13 (9.6)	3 (6.4)	8 (15.4)	2 (5.6)	0.198
Atrial fibrillation duration					
** **Duration (month)	14 ± 18	8 ± 14	13 ± 14	23 ± 24	0.001
** **Short lasting/long lasting/uncertain (n)	76/40/19	32/7/8	26/19/7	18/14/4	0.1064
CHA_2_DS_2_-VASc score	2.4 ± 1.5	2.3 ± 1.5	2.6 ± 1.5	2.3 ± 1.5	0.402
HAS-BLED score	1.3 ± 0.8	1.2 ± 0.8	1.4 ± 0.8	1.3 ± 0.6	0.352
LA volume (ml)	123 ± 29	111 ± 24	124 ± 28	138 ± 29	0.000
Left ventricular diastolic diameter (mm)	48 ± 5	47 ± 5	48 ± 5	50 ± 5	0.036
Left ventricular ejection fraction (%)	62 ± 10	62 ± 10	62 ± 10	62 ± 8	0.963

Values are presented as mean ± SD or as *n* (%). CPVI: circumferential pulmonary vein isolation; LA: left atrium; BMI: body mass index.

**Table 2:** Procedural parameters.

	Total (*n*=135)	CPVI responders (*n*=47)	LA substrate responders (*n*=52)	Nonresponders (*n*=36)	*P* value
Ablation time (sec)					
** **Total ablation time (sec)	2542 ± 783	1981 ± 566	2860 ± 736	2813 ± 702	<0.001
** **RPV ablation time (sec)	808 ± 249	813 ± 255	805 ± 260	809 ± 228	0.984
** **LPV ablation time (sec)	897 ± 272	955 ± 281	851 ± 249	911 ± 294	0.259
** **LA ablation time (sec)	812 ± 384		777 ± 403	862 ± 355	0.319
Fluoroscopy time (min)	23 ± 7	22 ± 7	22 ± 7	25 ± 7	0.084
Fluoroscopy dose (mGy)	303 ± 152	287 ± 132	305 ± 179	323 ± 133	0.561
Saline (ml)	1012 ± 277	804 ± 224	1162 ± 257	1065 ± 195	<0.001
LAA baseline AFCL (ms)	148 ± 21	159 ± 23	145 ± 16	138 ± 18	<0.001
LAA maximal AFCL (ms)	186 ± 25	213 ± 25	185 ± 20	172 ± 17	<0.001

Values are presented as mean ± SD. RPV: right pulmonary vein; LPV: left pulmonary vein; LA: left atrium; LAA: left atrial appendage; AFCL: atrial fibrillation cycle length.

## Data Availability

The data used to support the findings of this study are included within the article.
